# *Aspergillus terreus* spondylodiscitis following acupuncture and acupotomy in an immunocompetent host: case report and literature review

**DOI:** 10.3389/fcimb.2023.1269352

**Published:** 2024-01-04

**Authors:** Yufei Jin, Xiang Yin

**Affiliations:** Department of Spine Surgery, Daping Hospital, Army Medical University, Chongqing, China

**Keywords:** *Aspergillus terreus*, spondylodiscitis, acupuncture, acupotomy, allogeneic fibula ring

## Abstract

*Aspergillus terreus* is a fungus responsible for various infections in human beings; however, spine involvement is uncommon. Herein, we report a case of *A*. *terreus* spondylodiscitis following acupuncture and acupotomy in an immunocompetent Chinese patient. Admission lumbar magnetic resonance imaging (MRI) revealed infection at the L4/5 level without significant vertebral destruction. After unsuccessful symptomatic and anti-tuberculosis treatments, *A. terreus* was identified through culture, microscopy of isolate, histological examination and VITEK system. Intravenous voriconazole was then given; however, the patient’s spinal condition deteriorated rapidly, resulting in evident destruction of the L4/5 vertebral bodies. Surgeries including L4/5 intervertebral disc debridement, spinal canal decompression, posterior lumbar interbody fusion (PLIF) with allogeneic fibula ring fusion cages, and posterior pedicle screw fixation were then performed. Imaging findings at one-month and six-month follow-up suggested that the patient was successfully treated. This case highlighted two important points: firstly, although acupuncture and acupotomy are generally regarded as safe conservative treatments for pain management, they can still lead to complications such as fungal spinal infection. Therefore, vigilance is necessary when considering these treatments; secondly, PLIF with allogeneic fibula ring fusion cages may be beneficial for *A. terreus* spondylodiscitis patients with spinal instability.

## Introduction

*Aspergillus terreus*, a thermotolerant fungus belonging to the *Aspergillus* section *Terrei*, is commonly found in the environment. It can cause opportunistic infections, such as pneumonia (Modi et al., 2012), meningitis (Elsawy et al., 2015), endophthalmitis (Panigrahi et al., 2014), periprosthetic joint infection (Bartash et al., 2017), and cutaneous infection (Ozer et al., 2009), in both immunocompetent and immunocompromised patients. Spondylodiscitis is a rare but devastating disease that primarily affects the intervertebral disc and the adjacent vertebral bodies. *A. terreus* spondylodiscitis is even rare, with only ten reported cases (Seligsohn et al., 1977; Glotzbach, 1982; Brown et al., 1987; Grandière-Perez et al., 2000; Park et al., 2000; Maman et al., 2015; Comacle et al., 2016; Sohn et al., 2019; Takagi et al., 2019; Vithiya et al., 2023). In this report, we present a rare case of *A*. *terreus* spondylodiscitis following acupuncture and acupotomy in an immunocompetent Chinese patient. It’s worth noting that, posterior lumbar interbody fusion (PLIF) with allogeneic fibula ring fusion cages was firstly used to treat this kind of infectious spondylodiscitis.

## Case description

**Figure 1:**
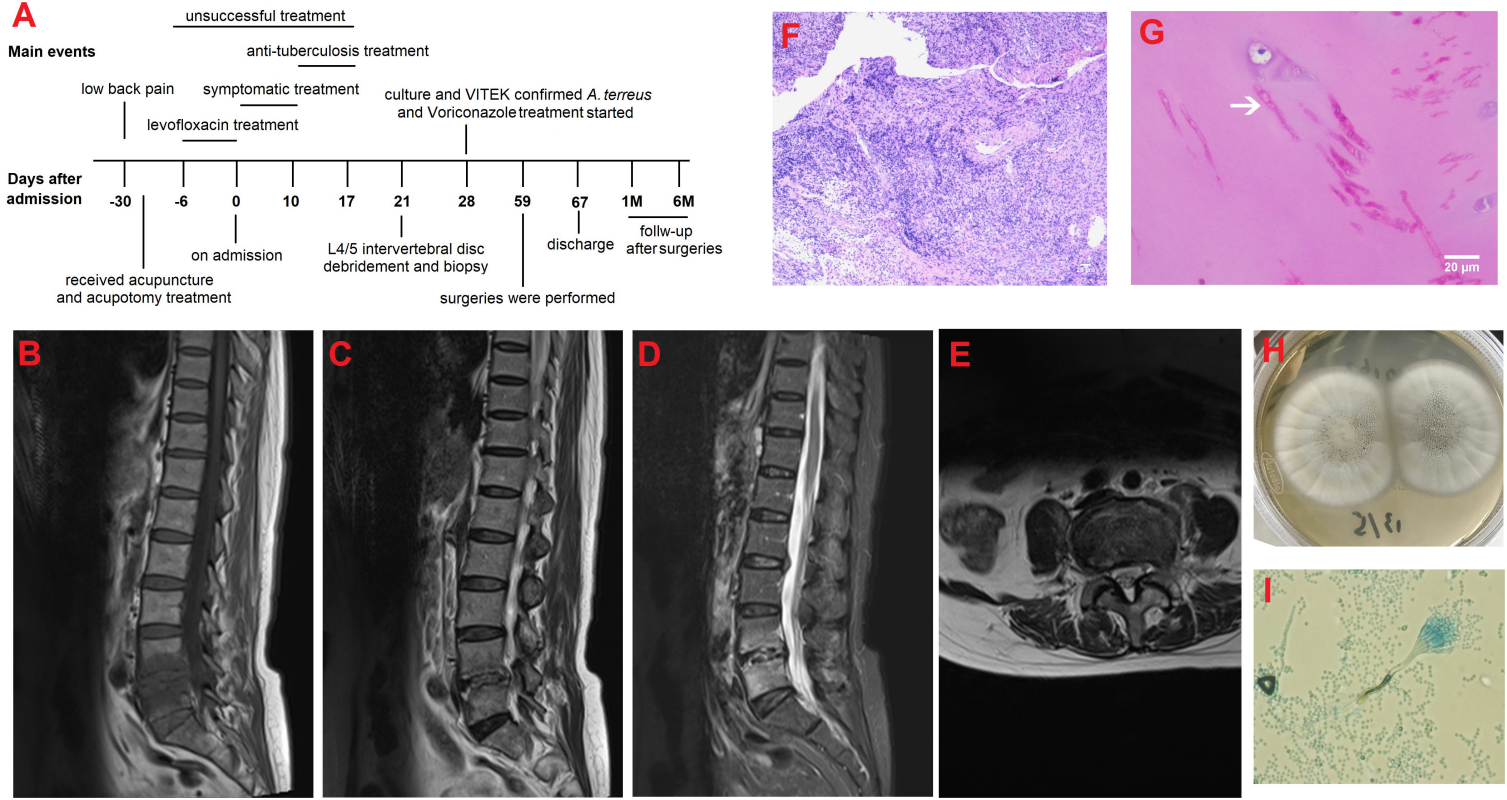
**(A)** The clinical timeline of the patient; **(B)** Lumbar sagittal T1-weighted MRI image at admission showed hypointensity of L4/5 vertebral bodies and intervertebral disc; **(C)** Lumbar sagittal T2-weighted image and **(D)** T2-weighted fat-suppression image at admission showed hyperintensity of L4/5 vertebral bodies and intervertebral disc; **(E)** Axial T2-weighted image at the level of L4/5 showed paravertebral soft tissue swelling and slight paraspinous abscess; **(F)** H&E staining showed massive inflammatory cells infiltration with some degree of purulent inflammation and necrosis (scale bar=20μm; magnification=10×); **(G)** Periodic acid–Schiff staining revealed septate fungal hyphae in the cartilage area; arrow indicates fungal hyphae (scale bar=20μm; magnification=40×); **(H)** Morphology features observed on the Sabouraud dextrose agar at 28°C after 5 days of incubation; and **(I)** Microscopic image of the subculture of the isolate following lactophenol cotton blue staining (magnification, 400×).

In October 2019, a 58-year-old female presented to our hospital due to recurrent low back pain. One month prior to admission, the patient suffered severe low back pain after physical labor work, with limited movement and difficulty in squatting, which were relieved after acupuncture and acupotomy (also called needle knife) treatment in a local clinic. Remarkably, her low back pain symptoms recurred repeatedly later. Magnetic resonance imaging (MRI) examinations in outside hospital showed multiple conditions such as lumbar degeneration, hyperostosis, endplate inflammation, lumbar disc herniation, and deep back fasciitis. Her abnormal laboratory findings [(white blood cells of 10.19×10^9^/L (normal range: 4-10×10^9^/L), platelet count of 313×10^9^/L (normal range: 100-300×10^9^/L), lymphocyte percentage of 14.2% (normal range: 20-40%), neutrophil percentage of 79.9% (normal range: 50-70%), neutrophil count of 8.14×10^9^/L (normal range: 1.8-6.3×10^9^/L), C-reaction protein (CRP) of 36.4 mg/L (normal range: 0-10 mg/L), erythrocyte sedimentation rate (ESR) of 89 mm/h (normal range: 0-20 mm/h), alkaline phosphatase of 158.1 U/L (normal range: 50-135 U/L), and albumin of 35.4 g/L (normal range: 40-55 g/L)] were far from being under control after 6 days of levofloxacin treatment. The patient was then admitted to the rheumatology and immunology department of our hospital (**Figure 1A**).

On admission physical examination showed obvious tenderness to percussion, activity limitation, and euesthesia. Laboratory findings indicated elevated CRP of 29.8 mg/L, fibrinogen of 5.74 g/L, interleukin-6 (IL-6) of 630.07 pg/ml (normal range: 0-7 pg/ml), and ESR of 101.0 mm/h. Her albumin was 34.8 g/L. Lumbar MRI at our hospital revealed abnormalities in the L4/5 vertebral bodies, intervertebral disc and surrounding soft tissues (**Figures 1B–E**). After 10 days of unsuccessful symptomatic treatment with anti-inflammatory and analgesic drugs, the patient was then transferred to the spine surgery department of our hospital for further treatment. Laboratory tests consistently showed inflammation (CRP of 47.0 mg/L, neutrophil count of 6.45×10^9^/L, IL-6 of 649.24 pg/ml, and ESR of 103.0 mm/h) and decrease in albumin (25.9 g/L), suggesting the possibility of tuberculosis infection. Thus, anti-tuberculosis treatment and strengthening nutrition intervention were given to the patient.

One week later, laboratory tests consistently showed inflammation (CRP of 37.0 mg/L, D dimer of 282.00 ug/L, white blood cells of 15.01×10^9^/L, neutrophil percentage of 80.2%, and ESR of 92.0 mm/h) and decrease in albumin (35.3 g/L). The poor effects of anti-tuberculosis treatment indicated a former wrong diagnosis of tuberculosis infection.

Four days later, L4/5 intervertebral disc debridement was performed with the assistance of lateral transforaminal endoscope. Purulent tissues were noticed in the L4/5 intervertebral space during operation. L4/5 disk space materials were biopsied for culture and histological examination. The symptoms of low back pain were alleviated after operation but aggravated two days later. H&E staining showed massive inflammatory cells infiltration with some degree of purulent inflammation and necrosis (**Figure 1F**). Periodic acid–Schiff staining revealed septate fungal hyphae (**Figure 1G**). One week after operation, biopsy culture (**Figure 1H**) and microscopy of isolate (**Figure 1I**) revealed *A. terreus*, which was also identified in a subculture of the isolate using the VITEK matrix-assisted laser desorption ionization time of-flight mass spectrometry (MALDI-TOF MS) system (BioMe´rieux, France); in addition, her galactomannan (GM) testing of biopsy specimen was positive with a value of 0.35. Based on the culture, microscopy of isolate, histological examination and VITEK system results, the patient was diagnosed as *A. terreus* spondylodiscitis and intravenously treated with voriconazole at a loading dose of 300 mg every 12 h on Day-1 followed by 200 mg every 12 h.

Ten days after antifungal therapy, her inflammatory indictors (CRP of 11.78 mg/L, IL-6 of 69.77 pg/ml, white blood cells of 8.21×10^9^/L, and ESR of 87 mm/h), low back pain and left lower limb pain were significantly improved than treatment before; however, repeated MRI and computerized tomography (CT) revealed aggravation of local vertebral destruction (**Supplementary Figures 1A–G**). Since her inflammation was alleviated after voriconazole treatment, conservative medical treatment was continued.

**Figure 2:**
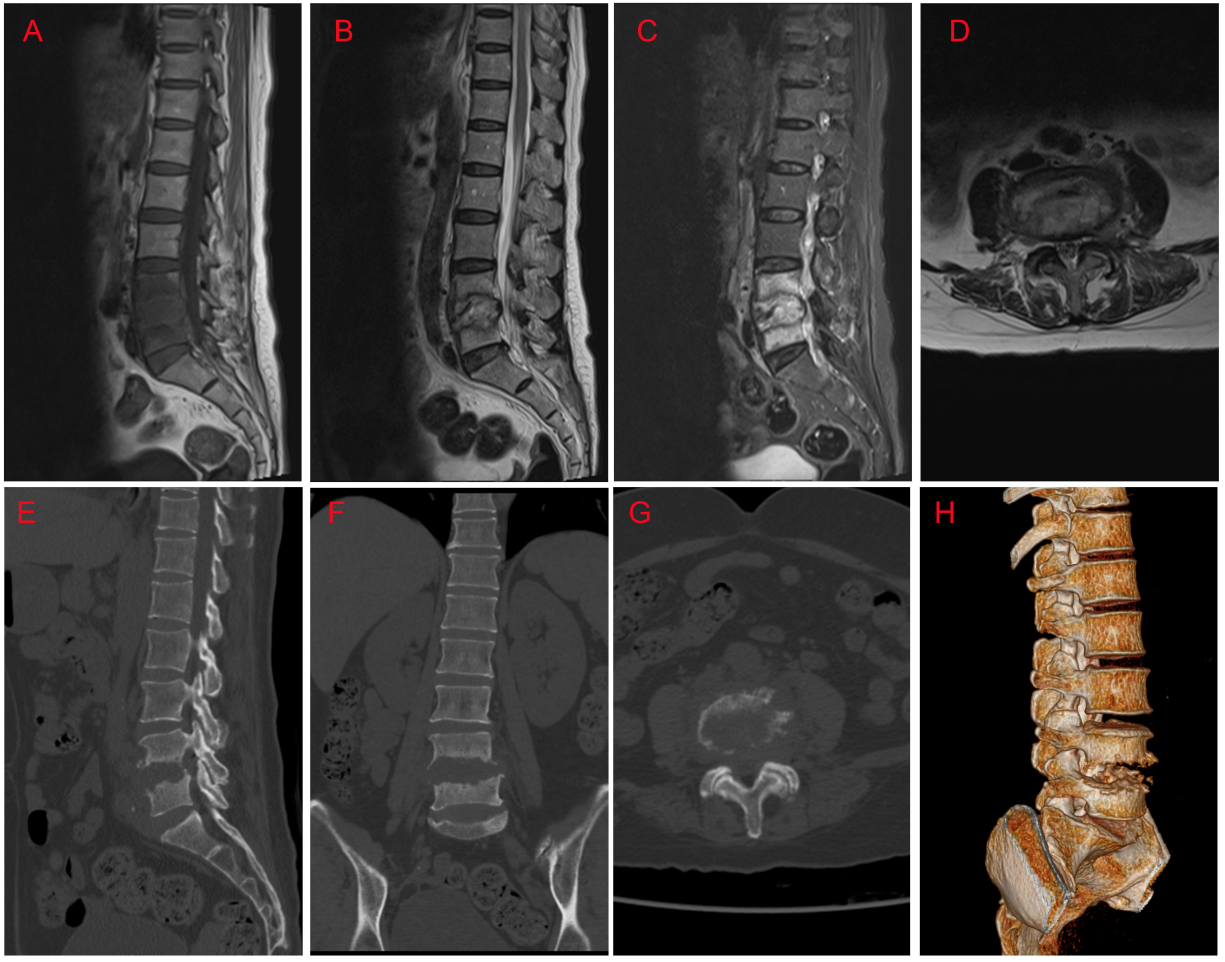
MRI and CT findings at approximately 24 days after antifungal therapy. **(A)** Sagittal T1-weighted image; **(B)** Sagittal T2-weighted image; **(C)** Sagittal T2-weighted fat-suppression image; **(D)** Axial T2-weighted image at the level of L4/5; **(E)** Sagittal CT image; **(F)** Coronal CT image; **(G)** Axial CT image; **(H)** Preoperative CT three-dimensional reconstruction image.

**Figure 3:**
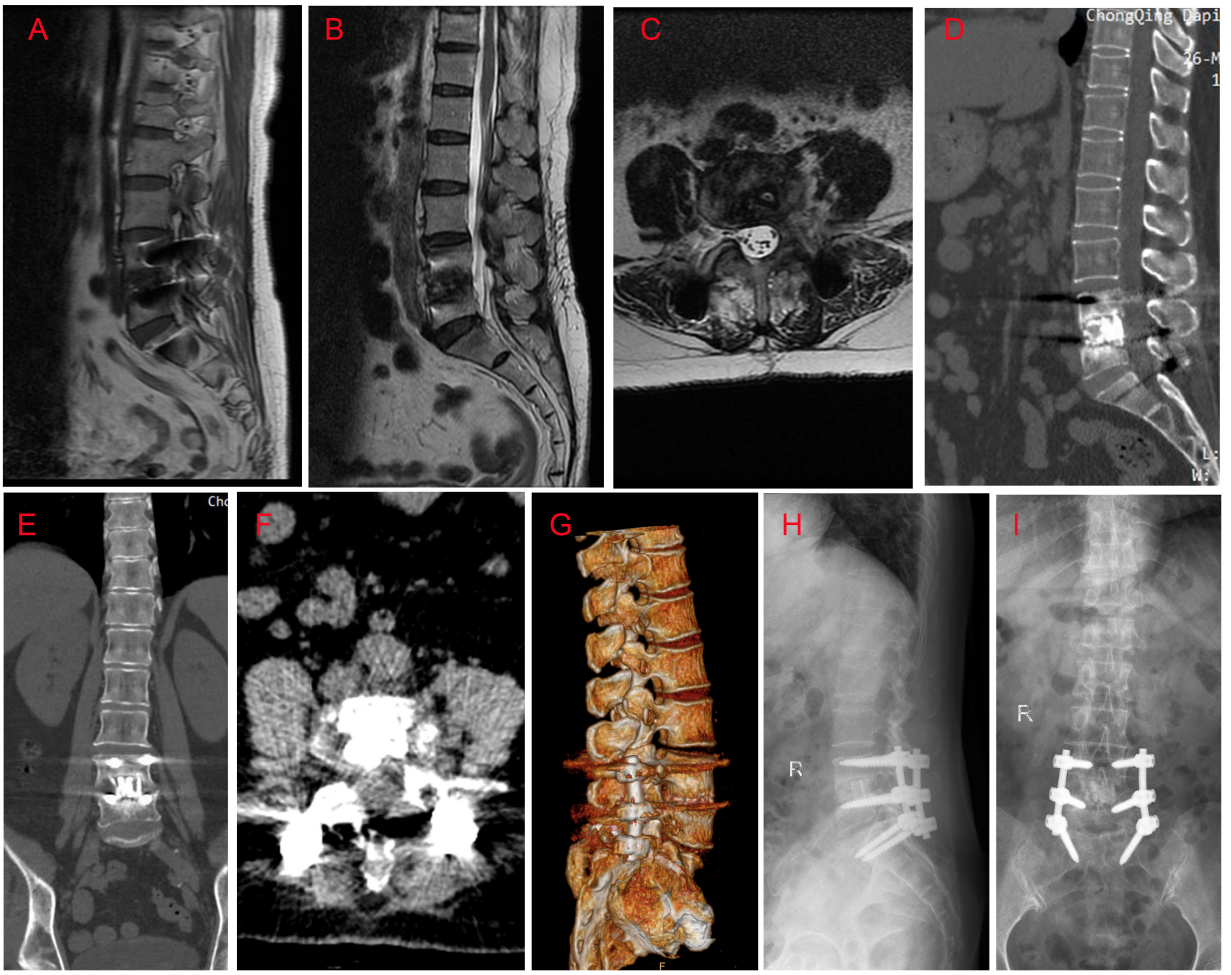
MRI, CT and X ray findings at six month follow-up. **(A)** Sagittal T1-weighted image; **(B)** Sagittal T2-weighted image; **(C)** Axial T2-weighted image at the level of L4/5; **(D)** Sagittal CT image; **(E)** Coronal CT image; **(F)** Axial CT image; **(G)** Lateral X ray image; **(H)** Anteroposterior X ray image; **(I)** CT three-dimensional reconstruction image. Imaging findings revealed that: the pedicle screws and cages were inserted in the right place, without cracked or displacement; there was no evidence of spinal infection; a certain level of L4/5 interbody fusion was observed.

Twenty-four days after antifungal therapy, although her inflammatory indictors (CRP of 7.52 mg/L, IL-6 of 69.86 pg/ml, white blood cells of 8.21×10^9^/L, and ESR of 29 mm/h) were further declined, her L4/5 vertebral bodies’ destruction still existed (**Figures 2A–H**). One week later, surgeries including L4/5 intervertebral disc debridement, spinal canal decompression, PLIF with allogeneic fibula ring fusion cages, and posterior pedicle screw fixation were successfully performed under general anesthesia (**Supplementary Figures 2A–H**). Antifungal therapy was continued after surgeries. Eight days after surgeries, the patient’s wound healed well and she was then discharged from our hospital. Imaging findings at one-month (**Supplementary Figures 3A–H**) and six-month (**Figures 3A–I**) follow-up demonstrated that the patient was successfully treated.

## Discussion

*A. terreus* is a saprophytic fungus that found in a variety of habitats, such as soil, dust, and compost heaps. While it is known to cause different types of infections, spine involvement is uncommon. To the best of our knowledge, only ten cases of *A. terreus* spondylodiscitis have been reported in humans (Seligsohn et al., 1977; Glotzbach, 1982; Brown et al., 1987; Grandière-Perez et al., 2000; Park et al., 2000; Maman et al., 2015; Comacle et al., 2016; Sohn et al., 2019; Takagi et al., 2019; Vithiya et al., 2023). The clinical characteristics of these reported cases and the current case are summarized in **Table 1**. Briefly, *A. terreus* spondylodiscitis occurred predominantly in males (9/11, 81.8%), without an age preponderance (range from 12 to 74 years old). The infection affected lumbar spine in 5 cases (45.5%), thoracic spine in 4 cases (36.4%), lumbar and thoracic spine in one case (9.1%), and lumbar spine and sacral section in one case (9.1%). All patients experienced back pain (100%), while four cases (36.4%) also presented with fever. *A. terreus* spondylodiscitis can occur in both immunocompetent and immunocompromised hosts. Surprisingly, the majority of reviewed cases (7/11, 63.6%) in this study were immunocompetent patient. As for the antifungal therapy, amphotericin B (AmB) was used as monotherapy in 4 cases (36.4%) and voriconazole in 5 cases (45.5%). Both the combination of AmB and itraconazole and the combination of voriconazole and caspofungin was reported in 1 case (9.1%).

**Table 1:** Clinical features of 11 reviewed *Aspergillus terreus* spondylodiscitis cases.

Author/year	Sex/age	Location	Symptoms	Immune status	Probable mechanism of infection	Predisposing factor (s)	Antifungal therapy	Surgery	Outcome
Seligsohn et al., 1977	F/42	L1-2	Low back pain	Immunocompetent	Hematogenous	IV drug abuse, alcoholism, liver cirrhosis, back injury	AmB	No	Recovered
Glotzbach, 1982	M/71	L2-3	Low back pain, fever	NM	Contiguous	Aortofemoral bypass surgery	AmB	No	Died
Brown et al., 1987	M/30	T6-7	Thoracic back pain, fever	Immunocompetent	Hematogenous	IV drug abuse	AmB	Yes	Recovered
Grandière-Perez et al., 2000	M/40	L3-4	Low back pain	Immunocompromised	Contiguous	AML, IPA	AmB, Itra	Yes	Died because of AML
Park et al., 2000	M/37	L3-S1	Low back pain	Immunocompromised	Contiguous	ALL	AmB	Yes	Recovered
Maman et al., 2000	M/67	L1-2, T6-9	Back pain, fever	Immunocompetent	NM	NM	Vori	NM	NM
Comacle et al., 2016	M/20	T7-12	Back pain, weakness, weight loss, cough, fever	Immunocompetent	Direct inoculation	TB, motorbike accident	Vori, Casp	Yes	Vertebral sequelae
Takagi et al., 2019	M/74	T11-12	Back pain	Immunocompetent	Direct inoculation	Abdominal stab wound	Vori	Yes	Recovered
Sohn et al., 2019	M/12	L1-4	Back pain	Immunocompromised	Contiguous	aGVHD	Vori	No	Recovered
Vithiya et al., 2023	M/45	T9-12	Back pain	Immunocompetent	NM	NM	Vori	Yes	Recovered
The present case	F/58	L4-5	Low back pain	Immunocompetent	Direct inoculation	None	Vori	Yes	Recovered

L, lumbar; S, sacral; T, thoracic; IV, intravenous; AmB, amphotericin B; Itra, itraconazole; Casp, caspofungin; Vori, voriconazole; AML, acute myeloid leukemia; IPA, invasive pulmonary aspergillosis; ALL, acute lymphoblastic leukemia; TB, tuberculosis; aGVHD, acute graft versus host disease; NM, not mentioned.

As we know, *A. terreus* can cause spine infection through three mechanisms: 1) contiguous spread from an adjacent site of infection; 2) hematogenous dissemination, and 3) direct inoculation (Comacle et al., 2016). Among all the reviewed cases, contiguous spread appears to be the predominant mode of transmission. In the four cases reported by Glotzbach et al.(Glotzbach, 1982), Grandie`re-Perez et al. (Grandière-Perez et al., 2000), Park et al. (Park et al., 2000), and Sohn et al.(Sohn et al., 2019), the infection may have resulted from contiguous spread from an adjacent site of infection. Moreover, in two cases reported by Seligsohn et al. (Seligsohn et al., 1977), and Brown et al. (Brown et al., 1987), the infection may have originated from hematogenous dissemination. It is worth mentioning that, with the exception of two cases that did not mention the mechanism of infection (Maman et al., 2015; Vithiya et al., 2023), in the remaining three cases (including the present case), direct inoculation might have been the contributing factor (Comacle et al., 2016; Takagi et al., 2019). In the case reported by Comacle et al., the fungus infection possibly arose from direct inoculation after a motorbike accident (Comacle et al., 2016). Takagi et al. reported one case of spondylodiscitis that probablely occurred due to the direct inoculation of *A. terreus* after an abdominal penetrating injury (Takagi et al., 2019). Acupotomy is a treatment method that uses specially designed needle-scalpels, either reusable or disposable. It is similar to traditional acupuncture, but more invasive. In China and other countries, including Korea, acupuncture and acupotomy are commonly used conservative therapies for pain management (Zhang et al., 2017). Although these therapies are generally considered safe and effective, they can cause adverse events such as spinal infection (Bang & Lim, 2006; P. S. Kim & Hsu, 2004; Y. J. White, 2004; Kim et al., 2014; Tsai et al., 2020). For instance, Bang and Lim reported one case of paraplegia which was caused by spinal infection after acupuncture (Bang & Lim, 2006). Tsai et al. reported one case of cervical spinal epidural abscess following acupotomy, in which reusable materials were used (Tsai et al., 2020). In this case, the patient didn’t have any predisposing conditions such as tuberculosis, intravenous drug use, diabetes mellitus, chronic obstructive pulmonary disease, or a history of laminectomy. Her past history was remarkable for receiving acupuncture and acupotomy in a local clinic to treat low back pain. She claimed that the needles and needle-scalpels used in acupuncture and acupotomy were reusable rather than disposable, which increased the possibility of infection. Following acupuncture and acupotomy, her symptoms recurred repeatedly and deteriorated rapidly after admission. Based on all above-mentioned information, we hypothesized the infection of this patient may have resulted from direct inoculation following acupuncture and acupotomy.

Since only few cases of *A. terreus* spondylodiscitis have been reported in human, the frequency of this condition is far from clear; however, we speculated that it may be underestimated because some cases may go undetected or misdiagnosed as tuberculosis due to shared clinical features and risk factors (Vithiya et al., 2023). Traditionally, the diagnosis of *A. terreus* is mainly based on cultures, microscopy, and/or histopathology. In some cases, VITEK MALDI-TOF MS method has been proven to be beneficial for the accurate diagnosis of *A. terreus* infection (Shin & Kim, 2021; Jing et al., 2022). Nevertheless, one thing we should keep in mind is that VITEK system has some limitations, such as limited ability of identification of rarely isolated species due to its narrow-spectrum database, which may lead to misidentification (Shin & Kim, 2021). Thus, a combination of VITEK and other methods, such as molecular sequencing and morphological evaluation, will contribute to more accurate diagnosis of *A. terreus*. In this case, the patient was diagnosed as *A. terreus* spondylodiscitis based on her culture, microscopy, histopathology, and VITEK system results.

The management of *A. terreus* spondylodiscitis can be challenging and often requires extended antifungal therapy, with or without surgical intervention. In the past, AmB was the preferred agent for the treatment of *A. terreus* spondylodiscitis; however, the clinical use of this drug in *A. terreus* infection has been limited because most isolates of *A. terreus* developed an intrinsic resistance against AmB (Posch et al., 2018; Vahedi Shahandashti & Lass-Flörl, 2019). Voriconazole is currently the preferred antifungal drug for treating *A. terreus* spondylodiscitis due to its broad antifungal coverage, favorable safety profile, and effective bone penetration. Conservative medical treatment may be sufficient for patients who only experience pain without significant spinal instability or neural compression; however, if these symptoms are present, surgical interventions such as debridement, spinal cord decompression, fusion, and fixation should be considered (Sohn et al., 2019). In this case, antifungal therapy alone was used after *A. terreus* was identified through culture. However, about one month later, the patient’s spinal condition deteriorated rapidly, resulting in significant lumbar instability. To address this, a combination of antifungal therapy and surgical interventions were performed. Notably, PLIF combined with posterior pedicle screw fixation were performed in this case to promote spine interbody fusion. During PLIF, two allogeneic fibula ring fusion cages loaded with autologous bone fragments were inserted. The hollow fibular ring filled with autogenous bone fragments can not only increase the contact area of autogenous bone graft, but also improve the support strength of the fibular ring. In addition, the large effective support area matching the anatomical morphology of the end plate provides a good biological and mechanical environment for efficient bone fusion (Shi et al., 2013). After the procedures, the patient recovered well with good bone fusion. However, it is important to note that although the surgical treatment options, which involved PLIF with allogeneic fibula ring fusion cages, have shown promising outcomes in our patient, further evidence is required to support their widespread clinical use in treating this kind of infectious disease.

## Conclusions

In this report, we present the first case of *A*. *terreus* spondylodiscitis following acupuncture and acupotomy in an immunocompetent Chinese patient. Although acupuncture and acupotomy are widely used in China as conservative treatments and are generally regarded as safe and effective, they can still lead to complications such as fungal spinal infection. Therefore, vigilance is necessary when considering these treatments. In addition, PLIF with allogeneic fibula ring fusion cages may be beneficial for *A. terreus* spondylodiscitis patients with spinal instability.

## Data availability statement

The original contributions presented in the study are included in the article/**Supplementary Material**. Further inquiries can be directed to the corresponding author.

## Ethics statement

The studies involving humans were approved by the ethics committee of Daping Hospital. The studies were conducted in accordance with the local legislation and institutional requirements. The participants provided their written informed consent to participate in this study. Written informed consent was obtained from the individual(s) for the publication of any potentially identifiable images or data included in this article.

## Author contributions

YJ: Conceptualization, Data curation, Investigation, Methodology, Project administration, Writing – original draft. XY: Conceptualization, Investigation, Methodology, Supervision, Visualization, Writing – original draft, Writing – review & editing.
