# Supplementary material for: Aspergillus terreus spondylodiscitis following acupuncture and acupotomy in an immunocompetent host: case report and literature review
Source: Front Cell Infect Microbiol. 2024 Jan 4;13:1269352. doi: 10.3389/fcimb.2023.1269352 (PMC10794653; doi:10.3389/fcimb.2023.1269352)
Supplement: Supplementary file 1 [file Image_1.pdf]

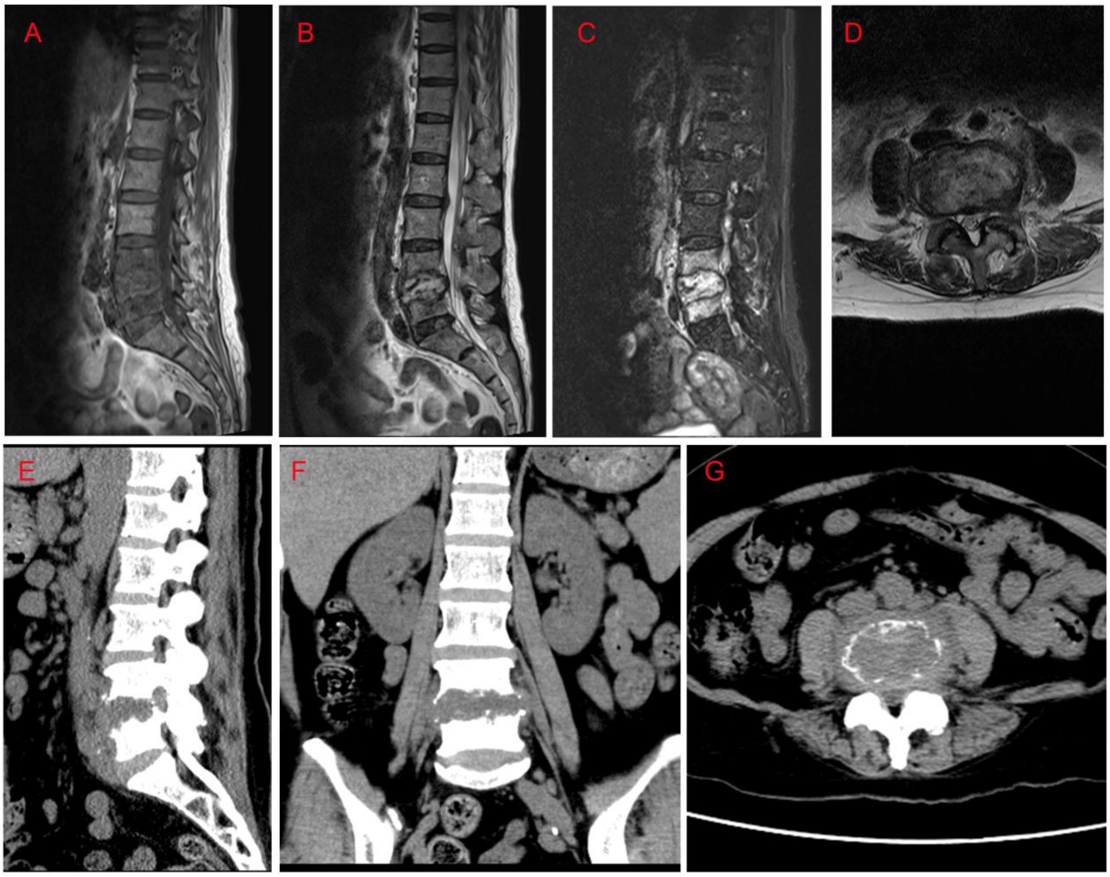

Supplementary Figure 1. MRI and CT findings at approximately 10 days after antifungal therapy revealed aggravation of spondylodiscitis. (A): Sagittal T1-weighted image; (B): Sagittal T2-weighted image; (C): Sagittal T2-weighted fat-suppression image; (D): Axial T2-weighted image at the level of L4/5; (E): Sagittal CT image; (F): Coronal CT image; (G): Axial CT image.
