# Supplementary material for: Aspergillus terreus spondylodiscitis following acupuncture and acupotomy in an immunocompetent host: case report and literature review
Source: Front Cell Infect Microbiol. 2024 Jan 4;13:1269352. doi: 10.3389/fcimb.2023.1269352 (PMC10794653; doi:10.3389/fcimb.2023.1269352)
Supplement: Supplementary file 3 [file Image_3.pdf]

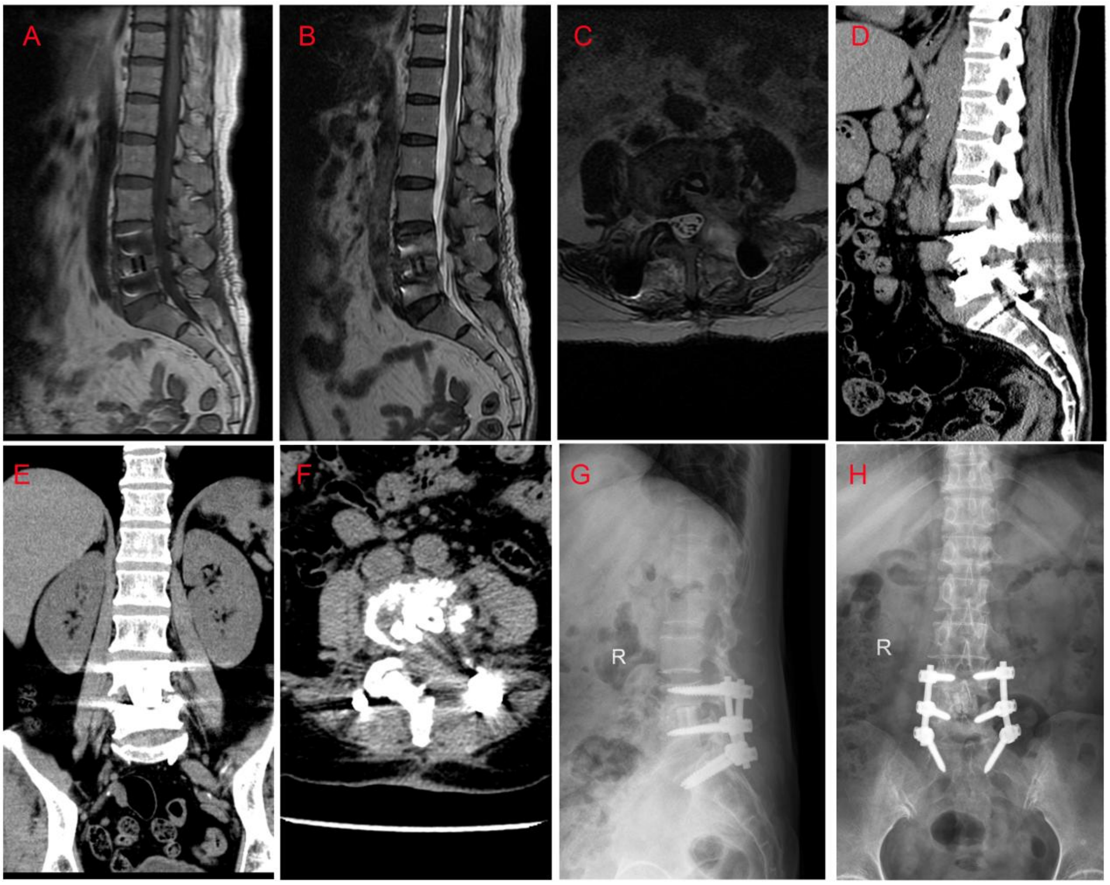

Supplementary Figure 3. MRI, CT and X ray findings at one month follow-up. (A): Sagittal T1-weighted image; (B): Sagittal T2-weighted image; (C): Axial T2-weighted image at the level of L4/5; (D): Sagittal CT image; (E): Coronal CT image; (F): Axial CT image; (G): Lateral X ray image; (H) Anteroposterior X ray image.
